# A Retrospective Career‐Long and Seasonal Study of Injury Patterns in 196 Elite Swimmers: The Role of Primary Discipline and Competitive Distance

**DOI:** 10.1111/sms.70256

**Published:** 2026-03-17

**Authors:** Sofie L. Nimb, Tobias Holst‐Christensen, Alexander Eggers, Marcel Rosenberg, Michael Kjaer, S. Peter Magnusson, Grith Højfeldt

**Affiliations:** ^1^ Institute of Sports Medicine Copenhagen, Department of Orthopaedic Surgery Copenhagen University Hospital Copenhagen Denmark; ^2^ Triton Lillestrøm Svømme‐ Og Triatlonklubb Skedsmokorset Norway; ^3^ Department of Clinical Medicine University of Copenhagen Copenhagen Denmark

**Keywords:** competitive distance, discipline, elite swimming, injury, injury epidemiology, shoulder injuries

## Abstract

Elite swimming is characterized by high training volumes, which increases the risk of overuse injuries. Detailed information on whether specific disciplines or competitive distance matters for the occurrence of injuries is sparse. This study aimed to describe the injury epidemiology among elite swimmers and to identify potential explanatory factors. We employed a retrospective, self‐reported design, with the known limitations of recall bias. In June 2024, a questionnaire was sent to elite swimming clubs across Denmark, Norway, and Sweden. A total of 220 swimmers completed the questionnaire describing training and injury characteristics in the 2023/2024 season and injury history from their entire swimming career. Throughout their careers, 128 out of 196 swimmers experienced at least one injury, resulting in a total of 183 injuries. We documented 113 injuries in 93 participants during the 2023/2024 season. For the 2023/2024 season, a total of 73 580 athlete exposures (AEs) were recorded, yielding an injury incidence of 1.54 injuries/1000 AEs. The shoulder was the most prevalent injury site (0.87 injuries/1000 AEs). Injury location appeared to differ across primary disciplines. When looking at overall injury incidence across disciplines, butterfly and breaststroke swimmers showed a slightly higher numerical injury incidence than other disciplines, although non‐significant (1.69 and 1.58 vs. 0.98 injuries/1000 AEs). Finally, freestyle sprinters had a higher injury incidence compared to freestyle long‐distance swimmers (rate ratio = 2.17, 95% CI 1.15–4.31). This indicates that both discipline and competitive distance play important roles in the risk for injury in elite swimming. However, due to the limitations of the study design, this should be interpreted with caution.

**Abbreviations**
AEs: athlete exposures
CI: confidence intervals
NCAA: National Collegiate Athletic Association
OR: odds ratio
REDCap: Research Electronic Data Capture
ROM: range of motion
RR: rate ratio
SD: standard deviation

## 1 Introduction

Swimming is among the most popular sports in many countries with a high number of active members (https://www.statistikbanken.dk/idrakt01, https://www.rf.se/forskning‐och‐statistik/statistik/idrottsrorelsen‐i‐siffror, https://www.idrettsforbundet.no/nyheter/2023/nokkeltall‐for‐norsk‐idrett‐2022/). Data exists on the prevalence and distribution of swimming‐related injuries. To date, studies of college‐level swimmers in the US have shown injury incidences varying from 1.48 to 5.55 injuries per 1000 athletic exposures (AE) [1, 2, 3, 4], while other studies have found that 43%–58% of swimmers sustain an injury over the course of a single season [3, 5, 6]. Due to its low‐impact nature, swimming is dominated by chronic sports injuries, and in general has a lower acute injury rate compared to other sports [5]. Elite swimming is characterized by extremely high training volumes with young adults training more than 16 h a week [7, 8]. Although swimmers compete across various disciplines, freestyle dominates training routines [7, 9, 10], which results in extremely high number of repetitions with up to 16 000 shoulder revolutions per week [11]. Unsurprisingly, different epidemiological studies have shown that the shoulder is the most injured body part [1, 2, 3, 4, 6, 12]. One study found that up to 91% of competitive swimmers reported shoulder pain within a single season [7]. This has given rise to the term *swimmer's shoulder* first described by Kennedy and Hawkins as a *common, painful syndrome of repeated shoulder impingement in swimmers* [13].

Although, several risk factors such as training volume, shoulder strength and mobility have been proposed as the underlying cause, its etiology remains unknown [8, 14, 15]. Some evidence suggests that freestyle swimmers are at the highest risk of injuries, particularly shoulder injuries [1, 2, 15]. These studies showed that most injuries occur during freestyle [1, 2]. However, because they did not consider the swimmers' primary discipline (i.e., the discipline in which they compete), our understanding of injury patterns across disciplines remains incomplete. There is thereby limited information on whether specific swim disciplines [butterfly, backstroke, breaststroke, freestyle] or competitive distance [sprinter, middle‐distance, long‐distance] matters for injury occurence [10]. Regarding competitive distance, Hill et al. [14] found low evidence linking distance to shoulder pain. Furthermore, only one epidemiological study investigated distance among elite swimmers and observed no difference in the occurrence of pain throughout an entire season. Previous research has therefore not addressed the overall injury epidemiology across primary discipline and competitive distance. This knowledge is highly important, as it can help identify high‐risk group, which is crucial for developing injury‐prevention strategies.

The primary objective of this study was to report the injury prevalence, incidence, and distribution of injuries among elite swimmers, and to fill the current knowledge gap by presenting these measures across disciplines. Secondly, it investigates the coupling of different explanatory factors such as specific swim disciplines and competitive distance with the injury incidence.

## 2 Materials and Methods

### 2.1 Study Design

This study was a retrospective cross‐sectional study using a questionnaire to gather data regarding self‐reported injuries. Injuries were recorded from the season of 2023/2024 and throughout participants' entire swimming career. This study design has known limitations of recall bias and will be discussed further in the discussion section. The study adhered to the guidelines of the latest *Declaration of Helsinki*. Based on Danish law, ethical board approval was not required as no biological material was collected.

### 2.2 Study Population

Participants recruited in this study were elite swimmers from Denmark, Sweden, and Norway. The authors (S.N., A.E., J.L., and M.R.) have close contacts to the elite swimming environment in the respective countries. They contacted the head coaches of elite swimming clubs, and the coaches distributed the questionnaire to the swimmers via e‐mail. Grith Højfeldt followed up with reminders to the clubs that didn't respond. The clubs that did not reply after the initial contact and a single reminder were not contacted further.

To be included in the study, participants had to be junior or senior swimmers (born before 2008). Furthermore, participants had to be actively competing as elite swimmers, defined as swimmers who train ≥ 4 times per week and compete at national championships. Participants were excluded if they were under the age of 15 and swam < 4 times per week. Eligible clubs included those with swimmers competing at least on a national level, with a large proportion of the clubs also having swimmers competing on an international level.

### 2.3 Data Collection

In June 2024 a questionnaire was sent out via the data‐base RedCap [16] to elite swimming clubs in Denmark, Sweden and Norway. The questionnaire was a bespoke instrument and collected data on participants' sex, age and swimming experience (years), along with primary discipline and competitive distance. Primary discipline was reported by the athletes at the time of inclusion (Supporting Information). For the last season (2023/2024), participants reported weekly number of swim sessions, average swim distance per session (meters) and weekly number of strength sessions. Participants also provided information on previous and/or current injuries, with information on location, duration, how it affected their training and lastly whether these injuries were evaluated by a doctor and how they were described. They reported injuries sustained both during the last season and throughout their entire swimming career (Supporting Information).

### 2.4 Variables and Definitions

**FIGURE 1:**
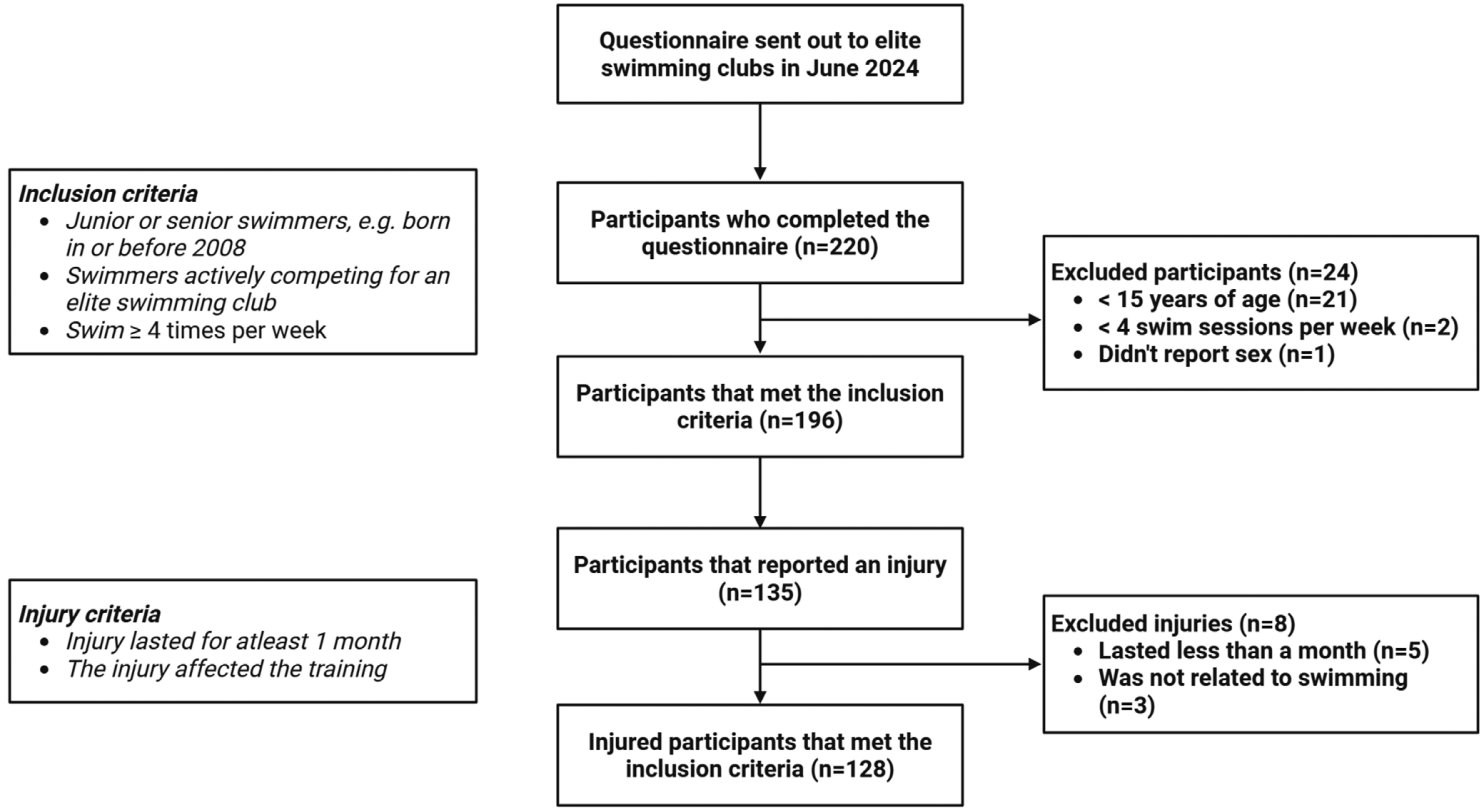
Inclusion and exclusion process for the study.

An injury was defined as a musculoskeletal problem, having lasted at least 1 month, and the injury had to affect training. This was clearly stated before answering the questionnaire (Supporting Information), and all answers were screened by study personnel. If the criteria (duration over 1 month and affecting training) were not fulfilled, the injury was not included in further analysis (Figure 1 for no. of exclusions). Injuries were categorized by body parts as follows: shoulder, elbow, back, hip, knee, ankle and “other region”. If an athlete answered “other region”, they had the option of specifying the injury. This is in accordance with the IOC consensus statement of recording and reporting of epidemiological data on injuries [17], as injuries were self‐reported.

An athlete exposure was defined as one swimming practice. Based on the number of training weeks, the participants' weekly sessions per week were extrapolated to AEs for the season 2023/2024. With the help of coaches, it was assumed that each participant held a break from training of 6.5 weeks between seasons. Competition was not included as an AE in this study.

Regarding primary discipline, participants were asked to identify their primary discipline at the time of inclusion, choosing from butterfly, backstroke, breaststroke, freestyle, and individual medley (from here on referred to as medley). Medley, a combination of all four main strokes, was considered an independent discipline in this study. If participants indicated multiple primary disciplines, they weren't included in the between discipline analysis but were included in all other analyses, including the binary logistic regression.

Additionally, distance refers to the elite swimmers' competitive distance. Participants were grouped based on their competitive distance. A ‘sprinter’ was defined as ≤ 100 m in their primary discipline, ‘middle‐distance’ as 200 m and ‘long‐distance’ as > 200 m.

### 2.5 Statistical Methods

To determine whether data could be pooled, we evaluated between‐country differences in age, primary discipline, and training characteristics. Based on this assessment, participants were pooled for all subsequent analyses, except for presentation of the participant characteristics (Table 1).

**TABLE 1:** Participant characteristics.

Demographics								
Country	Denmark		Sweden		Norway		Total	
Sex	Male	Female	Male	Female	Male	Female	Male	Female
*N*	51	37	37	35	18	18	106	90
Age (±SD)	19.8 ± 6.3	18.4 ± 3.3	18.0 ± 2.7	17.5 ± 1.9	18.4 ± 2.1	18.1 ± 2.4	18.9 ± 4.8	17.9 ± 2.7
Years of swimming (±SD)	12 ± 4	11 ± 4	11 ± 4	11 ± 3	11 ± 4	11 ± 2	11 ± 4	11 ± 3
Primary discipline^a^ (percentage are within nation and sex)								
Butterfly	11 (29%)	5 (16%)	5 (19%)	1 (5%)	2 (22%)	1 (11%)	18 (25%)	7 (11%)
Backstroke	4 (11%)	9 (28%)	4 (15%)	6 (29%)	1 (11%)	2 (22%)	9 (12%)	17 (27%)
Breast	4 (11%)	7 (22%)	6 (23%)	5 (24%)	3 (33%)	1 (11%)	13 (18%)	13 (21%)
Freestyle	14 (29%)	10 (31%)	11 (42%)	7 (33%)	3 (33%)	5 (56%)	28 (38%)	22 (35%)
Medley	5 (13%)	1 (3%)	0	2 (10%)	0	0	5 (7%)	3 (5%)
Event distance (percentage are within nation and sex)								
50 m	5 (10%)	1 (1%)	5 (14%)	7 (20%)	1 (6%)	0	7 (8%)	8 (9%)
100 m	13 (25%)	17 (46%)	16 (43%)	12 (34%)	8 (44%)	8 (44%)	37 (40%)	37 (41%)
200 m	18 (35%)	13 (35%)	10 (27%)	8 (23%)	6 (33%)	4 (22%)	34 (37%)	25 (28%)
400 m	9 (18%)	3 (8%)	3 (8%)	6 (17%)	3 (17%)	1 (11%)	15 (16%)	10 (11%)
800 m	2 (4%)	3 (8%)	1 (3%)	0	0	3 (33%)	3 (3%)	6 (7%)
1500 m	4 (8%)	0	2 (5%)	2 (6%)	0	2 (22%)	6 (7%)	4 (4%)
Sprint	18 (35%)	18 (47%)	21 (57%)	19 (54%)	9 (50%)	8 (44%)	44 (48%)	45 (50%)
Middle‐distance (%)	18 (35%)	13 (35%)	10 (27%)	8 (23%)	6 (33%)	4 (22%)	34 (37%)	25 (28%)
Long‐distance (%)	15 (29%)	6 (16%)	6 (16%)	8 (23%)	3 (17%)	6 (33%)	24 (23%)	20 (22%)
Training characteristics								
Swim trainings per week last season (±SD)	8.0 (1.2)	7.8 (1.3)	8.1 (1.5)	7.8 (1.3)	8.0 (1.0)	7.7 (1.0)	8.1 (1.3)	7.8 (1.2)
Swim training per week in hours	16.1 (3.98)	15.9 (4.7)	15.3 (3.1)	14.3 (3.9)	18.3 (8.3)	16.6 (4.3)	16.2 (4.8)	15.42 (4.37)
(±SD)								
Average distance per week last season in meters (±SD)^b^	40 200 (11321)	38 074 (8097)	41 229 (13400)	38 052 (12288)	37 134 (6755)	36 588 (8264)	40 004 (11424)	37 778 (9865)

^a^ 4 participants did not report primary discipline, 56 participants reported multiple primary disciplines and were excluded.

^b^ Average distance per week was calculated via participants reported swim trainings per week and average meters swum per session.

Prevalence estimates were calculated with 95% confidence intervals (CIs) using the Wilson score method. Injury incidence was calculated as injuries per 1000 AEs. Exact 95% Poisson CIs were calculated for each incidence estimate. Sex‐ and age‐group differences in incidence were evaluated using Poisson exact tests. To compare incidence across anatomical locations, the dataset was structured in long format, with each athlete contributing one row per location. Each row contained the same exposure, reflecting that all locations share the athlete's total exposure. A univariable Poisson regression with an offset for log (exposure) was used to estimate rate ratios (RRs), 95% CIs and *p*‐values with *elbow* as the reference value, as it had the lowest count of injuries.

For discipline and competitive distance differences were examined using a univariable Poisson regression model with the log (exposure) as an offset. Freestyle and long‐distance were the reference categories. RRs with 95% CIs and *p*‐values are reported. A subgroup analysis of freestyle swimmers across their competitive distance was calculated using the same approach.

A multivariable binary logistic regression model was used to examine associations between injury status (injured vs. non‐injured) during the previous season and swimmer characteristics. The swimmer characteristics were all included as predictors and included sex, age group, primary discipline, and competitive distance. If participants swam medley or reported multiple primary disciplines, they were collapsed into 1 discipline termed multi‐discipline. All predictors were coded as factors. Odds ratios (ORs) and 95% confidence intervals (CIs) were calculated. Analysis were conducted using RStudio, and figures were created using GraphPad Prism.

## 3 Results

### 3.1 Participant Characteristics

In total, 220 swimmers completed the questionnaire, out of which 102 (46%) were Danish, 74 (34%) Swedish, and 44 (20%) Norwegian. A flowchart depicting the inclusion and exclusion process is shown in Figure 1. Twenty‐one were excluded as they were under 15 years of age, two had under four swim sessions per week, and one participant did not report sex.

A total of 196 swimmers were included in this study: 88 (45%) were Danish, 72 (37%) Swedish, and 36 (18%) Norwegian. An overview of their characteristics is presented in Table 1. Participants trained an average of 7.9 (±1.2) times (AE) per week, totalling 15.8 (±4.6) hours. The mean distance per session was 4856 (±888) meters, translating to an estimated weekly total of 38 979 (±10 763) meters (Table 1). Most participants had freestyle as a primary discipline (*n* = 50 (37%)).

### 3.2 Seasonal Results

#### 3.2.1 Overall Epidemiology

**FIGURE 2:**
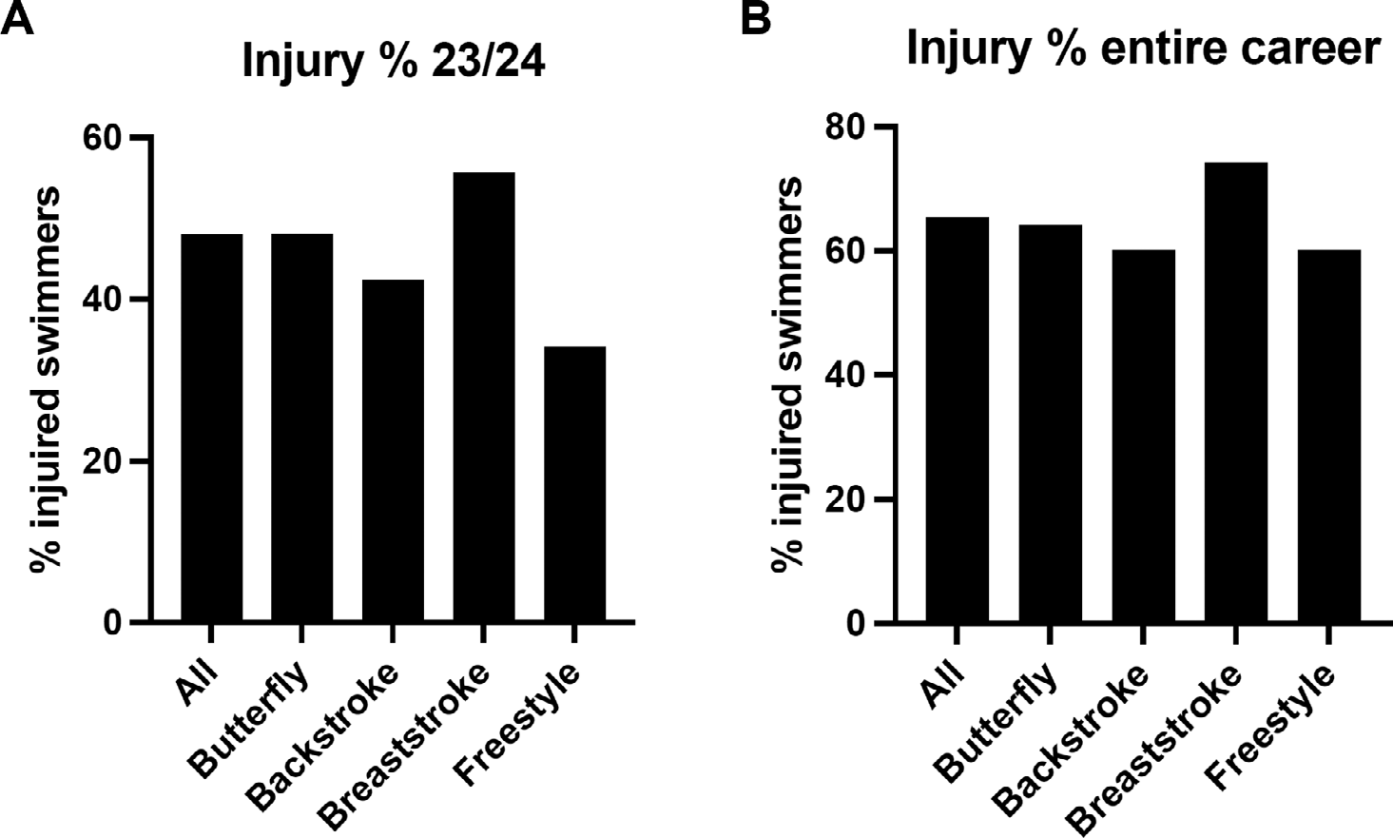
Prevalence of swimmers with an injury stratified by primary discipline. (A) season 23/24. (B) entire career.

In the 2023/24 season 93 participants suffered 113 injuries. This equates to a prevalence of 47.9% (95% CI 41.1%–54.9%) of the participants sustaining an injury (Figure 2A). When investigating the prevalence across disciplines, the percentage was highest among breaststroke with 55.6% (95% CI 37.3%–72.4%) and second highest for butterfly with 48% (95% CI 30%–66.5%).

A total of 73 580 AEs were documented, resulting in an overall injury incidence of 1.54 injuries per 1000 AEs (95% CI 1.26–1.85). Women reported 33 274 AEs and 54 injuries, equating to 1.62 injuries per 1000 AEs (95% CI 1.22–2.18), while men had 40 306 AEs and 59 injuries, resulting in 1.46 injuries per 1000 AEs (95% CI 1.14–1.89). The injury incidence was not significantly different between sexes (Figure S1) and age groups (Figure S2), and further analysis wasn't performed.

#### 3.2.2 Injury Location

**FIGURE 3:**
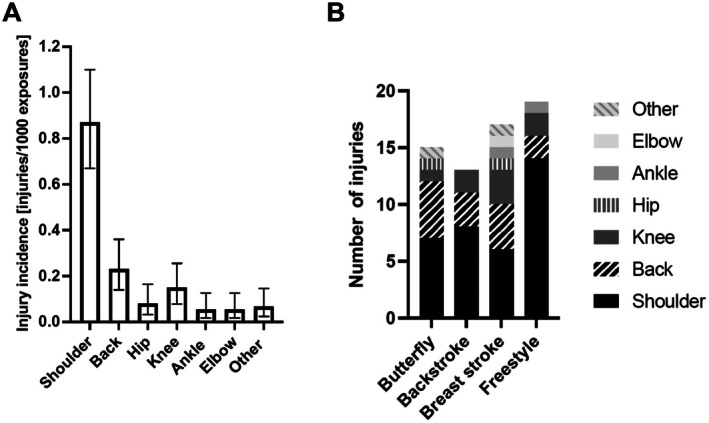
(A) Injury incidence [injuries/1000 AEs] by location, *n* = 196, 113 injuries in total 94 people had at least one injury. Bars represent incidence rates with 95% confidence intervals. Shoulder injuries showed the highest incidence, followed by back injuries. (B) No. of injuries divided into primary disciplines, *n* = 94. Other location included foot and neck injuries.

Regarding injury incidence across different anatomical locations, the shoulder was the most frequent location with 0.87 injuries/1000 AE (95% CI 0.67–1.10), followed by the back with an injury incidence of 0.23 injuries/1000 AEs (95% CI 0.14–0.36) (Figure 3A). Relative to elbow, shoulder (RR 16, 95% CI 6.6–52.6, *p* < 0.001) and back (RR 4.25, 95% CI 1.6–14.8, *p* = 0.009) demonstrated higher incidence rates. No other locations differed significantly. All estimates are provided in Table S1. Across all disciplines, the shoulder was the most prevalent injury location, followed by the back. No significant differences in injury location were observed across primary disciplines, but shoulder injuries tended to be more prevalent in freestyle whereas back pain was observed more frequently in breast and butterfly (Figure 3B).

#### 3.2.3 Primary Discipline and Competitive Distance

**FIGURE 4:**
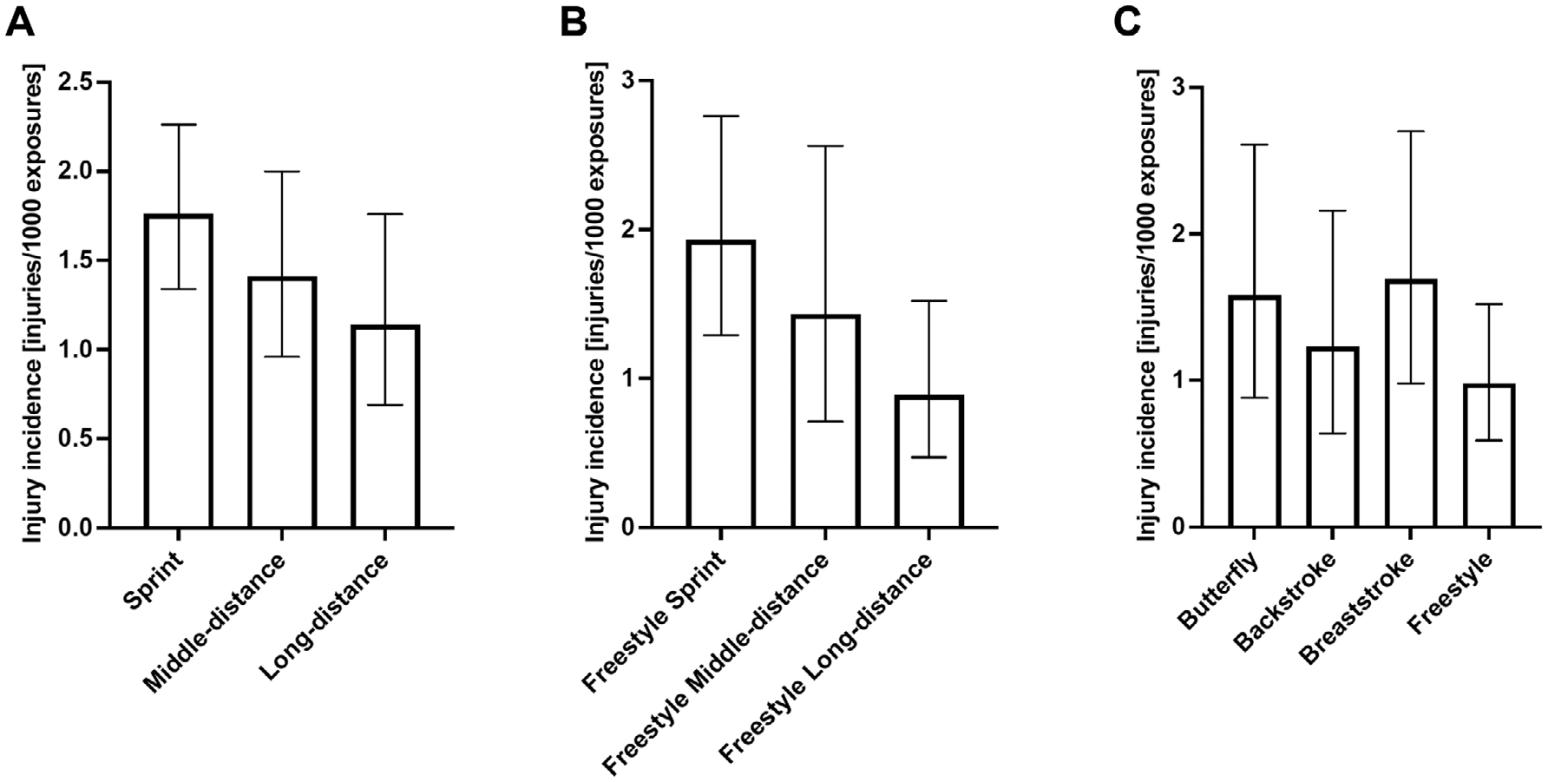
(A) Injury incidence [injuries/1000 AEs] stratified by competitive distance. Sprinters showed a tendency for higher injury incidence than long‐distance (RR 1.54 95% CI 0.95–2.62, *p* = 0.09). (B) Injury incidence [injuries/1000 AEs] for freestyle swimmers stratified by competitive distance. Freestyle sprinters showed a significantly higher incidence than long‐distance (RR 2.17 95% CI 1.15–4.31, *p* = 0.0203). (C) Injury incidence [injuries/1000 AEs] across all primary disciplines. 56 participants were excluded due to multiple primary disciplines. Four did not report primary discipline. Sprinter equals ≤ 100 m in their primary discipline, middle‐distance equals 200 m and long‐distance equals > 200 m as their competitive. Bars represent incidence rates with 95% confidence intervals.

When stratifying participants by their competitive distance (Figure 4a and Table S2) there was a tendency (*p* = 0.094) for sprinters (*n* = 94) to have a significantly higher injury incidence than long‐distance swimmers (*n* = 44) (1.76 vs. 1.14, RR 1.54 95% CI 0.95–2.62). Since most long‐distance swimmers listed freestyle as their primary discipline, a subgroup analysis for swimmers that had freestyle among their primary disciplines was conducted (Figure 4b and Table S3). Here, we found that freestyle sprinters (*n* = 42) suffered significantly (*p* = 0.0203) more injuries than freestyle long‐distance swimmers (*n* = 37) (1.92 vs. 0.89, RR 2.17 95% CI 1.15–4.31). Interestingly, long‐distance swimmers swam on average 9773 m more per week than sprinters (45 183 ± 10 235 (SD) meters vs. 35 409 ± 10 913 (SD) meters). Furthermore, we stratified the data by all primary disciplines (Figure 4c and Table S4). This revealed the highest injury incidence in breaststroke swimmers (*n* = 27) (1.69 injuries/1000 AEs), followed by butterfly (*n* = 25) (1.58 injuries/1000 AEs). No significant difference between disciplines were found.

#### 3.2.4 Factors Associated With Injury

When analyzing for differences in injury risk across sex, age group, distance group, and primary discipline, no significant differences were found (Table S5). To highlight, sprinters had an OR of 1.76 (95% CI 0.79–4.02) compared to long‐distance swimmers, and swimmers with more than one primary discipline had an OR of 1.66 (95% CI 0.64–4.40) compared to backstroke swimmers.

### 3.3 Career‐Long Results

Across their careers, 128 out of 196 swimmers experienced at least one injury, resulting in a total of 183 injuries. Of these 183 injuries, 132 (72%, 66% for men and 78% for women) were evaluated by a doctor. 65.3% (95% CI 58.4%–71.7%) of the swimmers sustained an injury, with 74% (95% CI 55.3%–86.8%) among breaststroke and 64% (95% CI 44.5%–79.7%) among butterfly (Figure 2B). There was no difference between sexes (Figure S3).

For the distribution of specific injury locations Table S6. The shoulder was the most common injury location across all groups.

## 4 Discussion

This study showed that elite swimmers in the 2023/2024 season had an overall injury incidence of 1.54 injuries/1000 AEs (95% CI 1.26–1.85). The most prevalent injury site was the shoulder, with a rate of 0.87 injuries/1000 AEs (95% CI 0.67–1.10). Butterfly and breaststroke swimmers had a numerical higher injury incidence compared to other disciplines. Finally, freestyle sprinters had a higher injury incidence compared to freestyle long‐distance swimmers, despite having a lower training amount (45 183 ± 10 235 (SD) meters vs. 35 409 ± 10 913 (SD) meters).

The injury incidence and percentage of injured swimmers in the present study is comparable to several previous studies on elite swimmers (1.48–5.55 injuries per 1000 AEs [1, 2, 3, 4, 10]). In the US, the National Collegiate Athletic Association (NCAA) reported incidence rates of 1.48, 1.56 and 1.78 injuries/1000 AEs [1, 2, 4], while Chase et al. [3] found a markedly higher incidence of 5.55 injuries/1000 AEs. It is noteworthy that the study by Chase et al. involved a smaller cohort that was followed prospectively, whereas most studies relied on either an injury surveillance approach [1, 2, 4] or a retrospective questionnaire similar to the approach we present [6, 10]. The prospective design allows for more detailed and consistent monitoring of injuries over time, where retrospective questionnaires can underestimate the incidence of injuries due to recall bias. Further, it should be noted that age groups differ between various studies [1, 2, 3, 4, 10]. Athletes from the NCAA corresponds to our senior swimmers, while the group of elite swimmers from Bak et al. [10] correspond to our junior swimmers. As we did not find a difference in injury incidence (Figure S2) or injury risk (Table S5) comparisons between different age groups can still be done, though they should be interpreted with caution.

In the current study, shoulder injuries accounted for 53% of all injuries, which is consistent with previous epidemiological findings [1, 2, 3, 4, 6, 10, 12]. This is possibly explained by the high training load, which has been shown to lead to supraspinatus tendinopathy in 69% of swimmers [7]. This is supported by Yanai et al. [18] who demonstrated that biomechanical impingement occurs during 24.8% of the freestyle stroke cycle. Yanai et al. showed a large variability among swimmers (13.1%–39.3%), which suggests that the wide variation in stroke biomechanics may predispose some swimmers, but not others. A risk that may increase as pain increases, as shoulder pain has been shown to alter muscle coordination in swimmers [19]. To date, no study has examined injury location in relation to the swimmer's primary discipline. In our population, shoulder injuries were the most frequent across all disciplines. However, all swimmers, regardless of their primary stroke, predominantly train freestyle [7, 9, 10], which may explain why shoulder injuries are frequent in swimmers across all disciplines.

When comparing freestyle sprinters with freestyle long‐distance, sprinters had a significantly higher risk with an injury rate of 1.92 injuries/1000 AEs, compared to long‐distance swimmers (RR 2.17 95% CI 1.15–4.31). This is even though freestyle sprinters, in our cohort, swam approximately 10 000 m less than long‐distance swimmers while at the same time training almost the same number of hours per week (15.2 ± 4.6 (SD) vs. 16.9 ± 3.4 (SD)). We therefore hypothesize that sprinters engage in more high‐intensity training requiring longer breaks between intervals, which may explain the higher injury risk. Yoma et al. [20] demonstrated that a high‐intensity swim session acutely reduces external‐rotation ROM, which could increase susceptibility to shoulder injury. Further, if sprinters train their specific discipline they will train at a faster pace, creating a larger load on their bodies which may further make them more prone to shoulder injuries. This suggests that training volume alone does not fully account for injury risk, and that stroke intensity is a contributing factor. Nevertheless, as mentioned by McKenzie et al. [15], the causal relationships between these risk factors and injury remain uncertain.

Studies investigating injury rates across disciplines show that most injuries occur during freestyle [1, 2]. However, these studies report which discipline is performed when the injury occurred, rather than the swimmers habitual primary discipline. Contradictory, McKenzie et al. [15] presents some evidence against freestyle as an independent risk factor for shoulder pain in competitive swimmers. In general, the evidence on injury risk across disciplines is sparse [21]. In our study, when looking at total number of injuries (Table S6), freestyle swimmers did suffer the highest amount of injuries but also constituted the largest proportion of swimmers. Regarding injury risk, no differences were found between disciplines (Table S5). However, we found that swimmers who reported butterfly and breaststroke as their primary discipline had a numerically higher injury incidence compared to the other disciplines. In addition to shoulder injuries, butterfly and breaststroke swimmers had a high number of back injuries. This is a well‐known problem due to the hyperextension of the lower back, which is exaggerated in these disciplines [5, 21, 22]. Our finding was not statistically significant, and 43 participants were excluded due to multiple primary disciplines, leading to small sample sizes per group. Thus, conclusions regarding elevated injury risk in these disciplines remain speculative.

The primary injury type in elite swimming is overuse injuries, due to the repetitive nature of the training [1, 2, 3, 4, 12]. This cyclic loading repeatedly stresses the same anatomical structures [7, 8], and although the shoulder is most frequently affected, other regions are similarly impacted [21]. Because our study could not consistently differentiate between injury type, we cannot determine whether specific regions were more prone to overuse injuries than others. Overuse injuries are known to be underestimated in epidemiologic studies that rely on time‐loss definitions [23], as athletes typically train despite symptoms. Despite this underreporting, overuse injuries remain clinically important because they can impair training for prolonged periods [8] and negatively affect performance [24].

While swimmers have one of the highest training volumes reported [25], it should be noted that the injury incidence remains low compared to other elite sports [26, 27]. In a Swedish population of elite youth athletes [26], an average injury incidence of 4.1 injuries/1000 h was found, despite training only 7–10 h per week, compared to our group training 15–16 h weekly. When looking at injury type, other non‐contact sports such as rowing [28] or softball also tend to have more overuse injuries, whereas contact sports show a higher amount of traumatic injuries [27]. Another low‐impact sport also known for its repetitiveness and high training volume is elite road racing [25]. Unsurprisingly, a cohort of elite road cyclists also suffer a large number of overuse injuries [29]. To reduce this risk at a younger age, the Danish Cycling Union have proposed age‐specific guidelines for weekly training hours (https://www.cyklingdanmark.dk/fileadmin/user_upload/Landevej_traeningsanbefalinger_2025.pdf). This may be worth considering in swimming, as many young athletes experience an injury early in their career.

Different preventive strategies have been proposed to reduce the overuse injuries in swimming [21]. Outside the pool, a combined strengthening, stretching, and manual therapy have been shown to reduce shoulder pain [30], while stroke technique modification can lower impingement risk in freestyle [18]. A more targeted and individualized training approach may be particularly beneficial for high‐risk groups. For example, long‐term individualized prevention programs have been effective in reducing injury incidence [5]. This may benefit butterfly and breaststroke swimmers, the two disciplines with the highest injury incidence in our study, who, in addition to shoulder injuries, also experienced back injuries. Further, it is possible that sprinters could benefit from a more targeted training strategy that prioritizes speed and technique over sheer volume. By reducing the training volume and tailoring sessions to the specific physiological and biomechanical demands of sprint performance, it may be possible to lower injury risk without compromising competitive outcomes. Future studies should explore whether such a training modification could improve both performance and athlete health in this population.

### 4.1 Limitations

In this study an injury was defined as one that affected the training and lasted at least 1 month. This deliberate criterion aimed to differentiate between clinically relevant injuries and the transient muscle soreness or discomfort that is a normal part of any elite training. Previous studies define an injury as having lasted for at least 1 day or required treatment from their coach or physio [1, 2, 3, 4, 6, 12]. A consensus statement by Mountjoy et al. [23] supports the use of a more lenient injury definition, particularly with respect to injury duration. Our approach therefore likely resulted in a lower reported injury incidence. However, because this study was based on a retrospective questionnaire to gather data regarding self‐reported injuries, which has the known risk of recall bias and potential for misclassification, a stricter definition of an injury is particularly appropriate. As Gabbe et al. [31] emphasized, recall accuracy diminishes as the detail required increases, making longer‐lasting injuries more reliably remembered in retrospective designs. Longer‐standing injuries also tend to have a larger impact on an athlete's career and are more likely to require medical intervention. This is supported by the fact that most injuries (72%) were assessed by a medical doctor.

Primary discipline may change over the course of a career, and we were unable to determine whether such changes occurred. Although discipline is stable within a single season, changes across longer periods may have introduced misclassification. Discipline‐specific results over the entire career should therefore be interpreted with caution (Table S1).

Small sample sizes across disciplines, combined with the exclusion of swimmers with multiple primary disciplines, led to small sample sizes per group. Thus, conclusions regarding elevated injury risk in these disciplines remain speculative. Finally, whether competitive distance reflects training intensity remains speculative, although this assumption seems highly reasonable based on the data presented in this study.

### 4.2 Perspectives

We found an injury incidence of 1.54 injuries/1000 AEs, as reported in other epidemiological studies. Secondly, we showed a numerically higher injury incidence in butterfly and breaststroke swimmers. The shoulder was the most common injury location overall and across disciplines. For freestyle swimmers, sprinters had a significantly higher injury rate despite training less. This indicates that both discipline and competitive distance play a role in injury risk for elite swimmers. However, due to the limitations of the study design, this should be interpreted with caution.

Moving forward, to avoid recall bias, it would strengthen our knowledge to collect data on primary discipline and competitive distance prospectively. This would further increase the level of detail, both regarding the injury and the injury setting (i.e., training vs. competition). Secondly, a biomechanical analysis of the forces exerted on the body, and particularly on the joints, during sprint vs. longer distance races would be valuable. Combining this with data on the intensity of training sessions for sprint vs. long‐ distance swimmers, could help clarify the higher injury incidence in sprinters. Lastly, a targeted training strategy for the different kinds of swimmers (e.g., sprinters vs. long‐distance) may benefit the high‐risk groups identified in this study.

## Author Contributions

All authors met the Vancouver criteria for authorship. S. Peter Magnusson, Michael Kjaer, Grith Højfeldt and Sofie L. Nimb contributed to the study conception and design. Grith Højfeldt, Sofie L. Nimb, Alexander Eggers and Marcel Rosenkilde performed data collection. Data analysis was performed by Sofie L. Nimb, Tobias Holst‐Christensen and Grith Højfeldt. The first draft of the manuscript was written by Tobias Holst‐Christensen, S. Peter Magnusson and Grith Højfeldt. All authors approved the final version.

## Funding

The authors have nothing to report.

## Ethics Statement

As the questionnaire was anonymous, no ethical approval was necessary. All participants consented to take part in the study while answering the questionnaire.

## Conflicts of Interest

The authors declare no conflicts of interest.

## Supporting information


**Data S1:** Supporting Information.

## Data Availability

The data that support the findings of this study are available on request from the corresponding author upon reasonable request.
