# Supplementary material for: A Retrospective Career‐Long and Seasonal Study of Injury Patterns in 196 Elite Swimmers: The Role of Primary Discipline and Competitive Distance
Source: Scand J Med Sci Sports. 2026 Mar 17;36(3):e70256. doi: 10.1111/sms.70256 (PMC12994441; doi:10.1111/sms.70256)

Supplementary material – Figures

**Figure S1 - Injury incidence [injuries/1000 AEs], stratified by sex. Women (n=90) = 1.62 (1.22-2.18), men (n=106) = 1.46 (1.14-1.89). RR = 1.11 (0.75-1.63), p=0.6368**

*
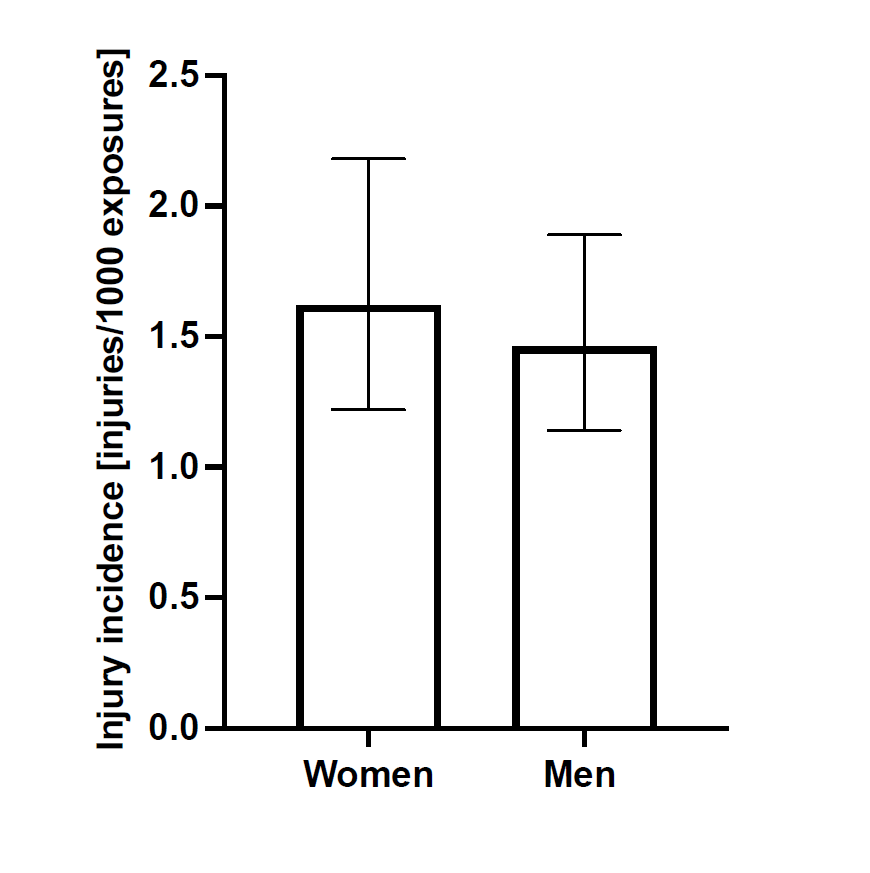
*

**Figure S2 – injury incidence [injuries/1000 AEs], stratified by age group. Junior (n=91) = 1.61 (1.21-2.11), senior (n=105) = 1.42 (1.08-1.84). RR = 1.14, (0.77-1.68), p=0.5063**


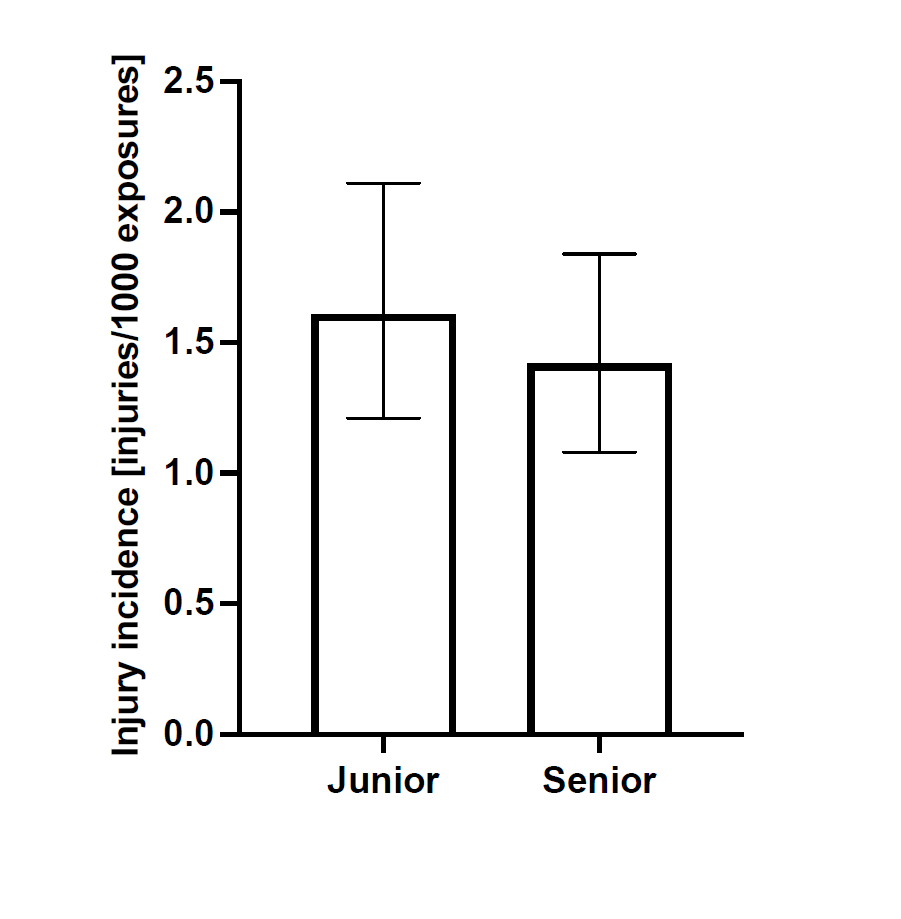


**Figure S3 - Percentage of swimmers with an injury, stratified by sex. A. season 23/24. B. entire career.**
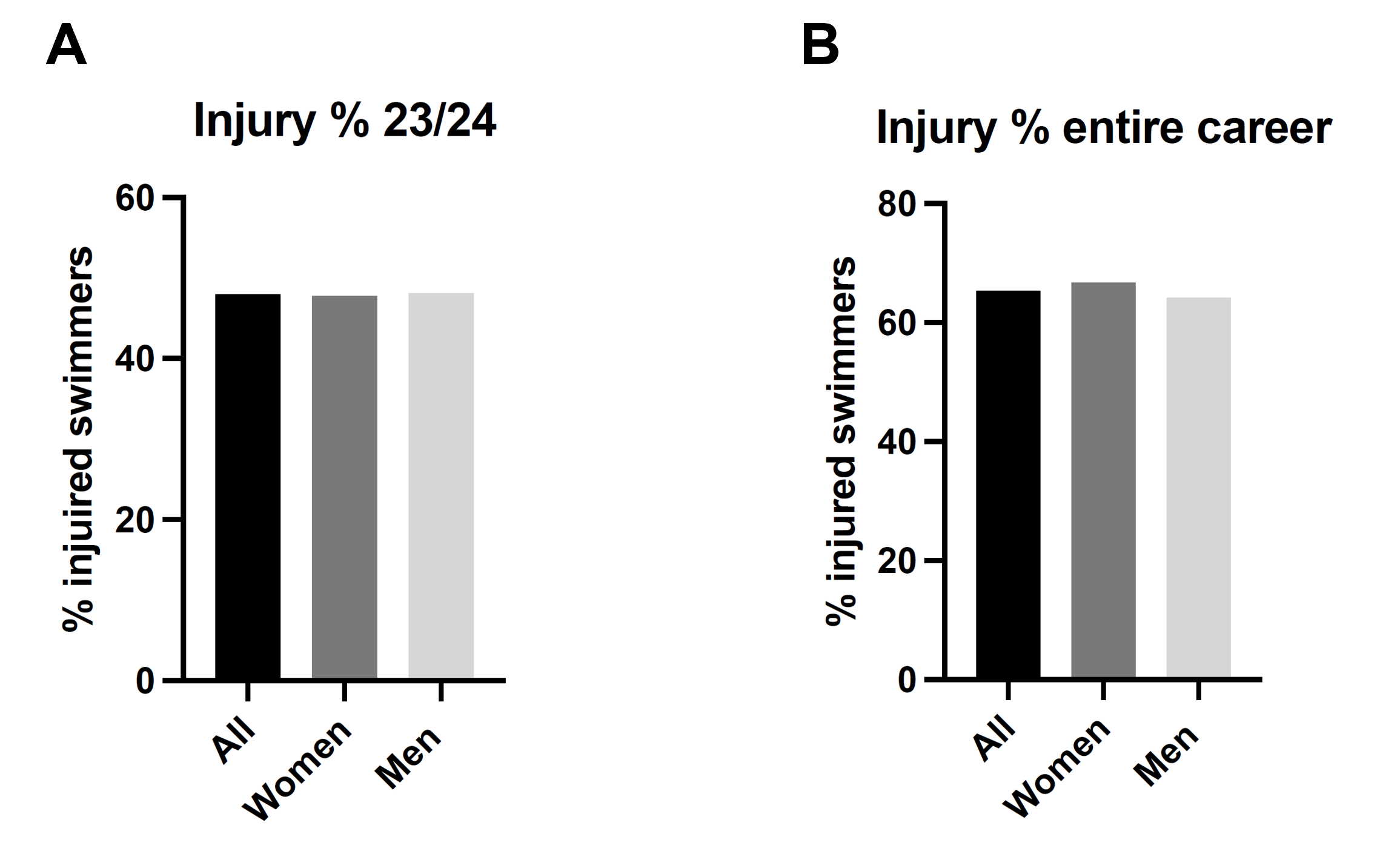

Supplement: Supplementary file 1 — Data S1: Supporting Information. [file SMS-36-e70256-s001.docx]
